# Supplementary material for: Varicella-zoster virus VLT-ORF63 fusion transcript induces broad viral gene expression during reactivation from neuronal latency
Source: Nat Commun. 2020 Dec 10;11:6324. doi: 10.1038/s41467-020-20031-4 (PMC7730162; doi:10.1038/s41467-020-20031-4)
Supplement: Supplementary file 1 — Supplementary information [file 41467_2020_20031_MOESM1_ESM.pdf]

Supplementary Information

Varicella-zoster virus VLT-ORF63 fusion transcript induces broad viral gene expression during reactivation from neuronal latency

Ouwendijk and Depledge et al.

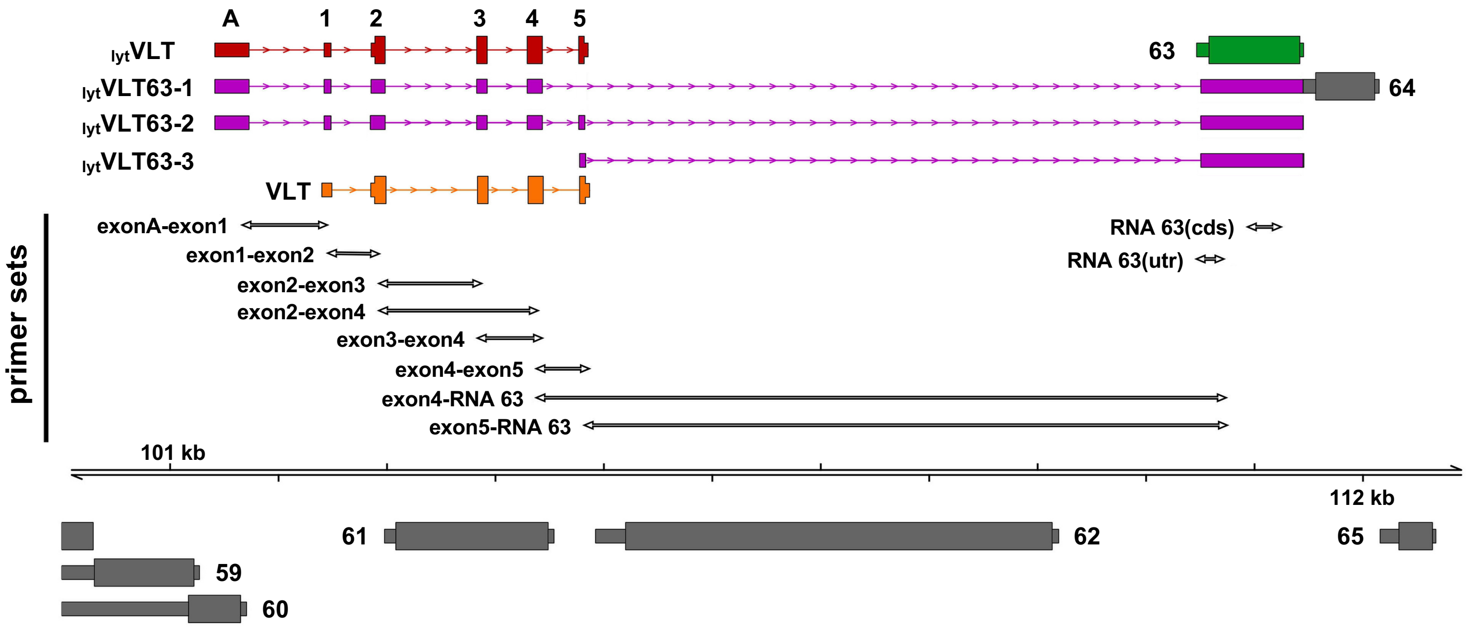

Supplementary Figure 1. Location of primer sets detecting transcripts from VLT and ORF63 loci. Schematics of the major transcripts from VLT and ORF63 loci are shown in following colors: lytic VLT isoform ($_{lyt}$VLT, red), canonical RNA 63 (63, light green), lytic VLT-ORF63 isoforms ($_{lyt}$VLT63-1/2/3, purple) and latent VLT isoform (VLT, orange). Additional canonical VZV transcription units present are shown in grey, with ORF numbers indicated. Wide boxes indicate canonical CDS domains and while thin boxes indicate UTRs, respectively. Location of primer sets (**Supplementary Table 1**) used for RT-qPCR analysis to detect transcripts from VLT and ORF63 loci are depicted by black arrows. ORF; open reading frame, CDS; coding sequence, UTR; untranslated region.

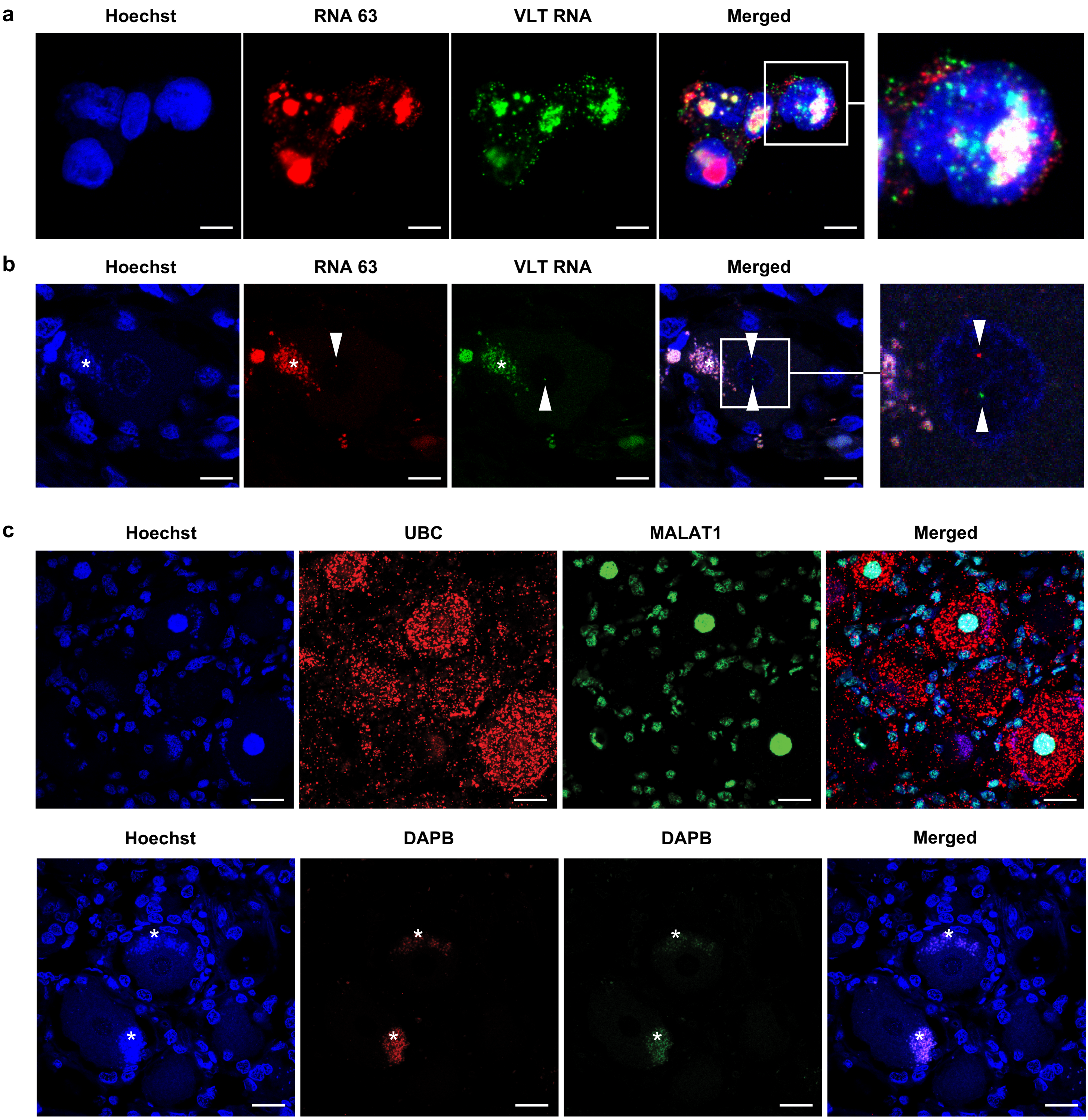

Supplementary Figure 2. Detection of VZV RNA 63 and VLT RNA by fluorescent multiplex in situ hybridization. Detection of VZV RNA 63 and VLT RNA by multiplex fluorescent in situ hybridization (mFISH) on **a** lytically VZV-infected ARPE-19 cells and **b** latently VZV-infected human trigeminal ganglia (TG). Representative images are shown for **a** n=2 independent experiments and **b** n = 7 human TG. **a, b** Right panels represent enlargements of area indicated by white box. **b** Rare detection of RNA 63 (red) and VLT (green) as discrete puncta in nuclei of human TG neurons. Asterisks indicate autofluorescent lipofuscin granules in neurons and arrowheads indicate RNA 63 and/or VLT mFISH signal. **c** Human TG were stained with mFISH using positive (UBC; human ubiquitin C [red] and MALAT1; human metastasis associated lung adenocarcinoma transcript 1 [green]) and negative control probes (DAPB; bacterial dihydrodipicolinate reductase, green and red) to demonstrate specificity of the mFISH assay and RNA integrity of the tissue assayed. Nuclei were stained with Hoechst (blue). Scale bars: 10 μm for **a** and **b**, and 20 μm for **c**.

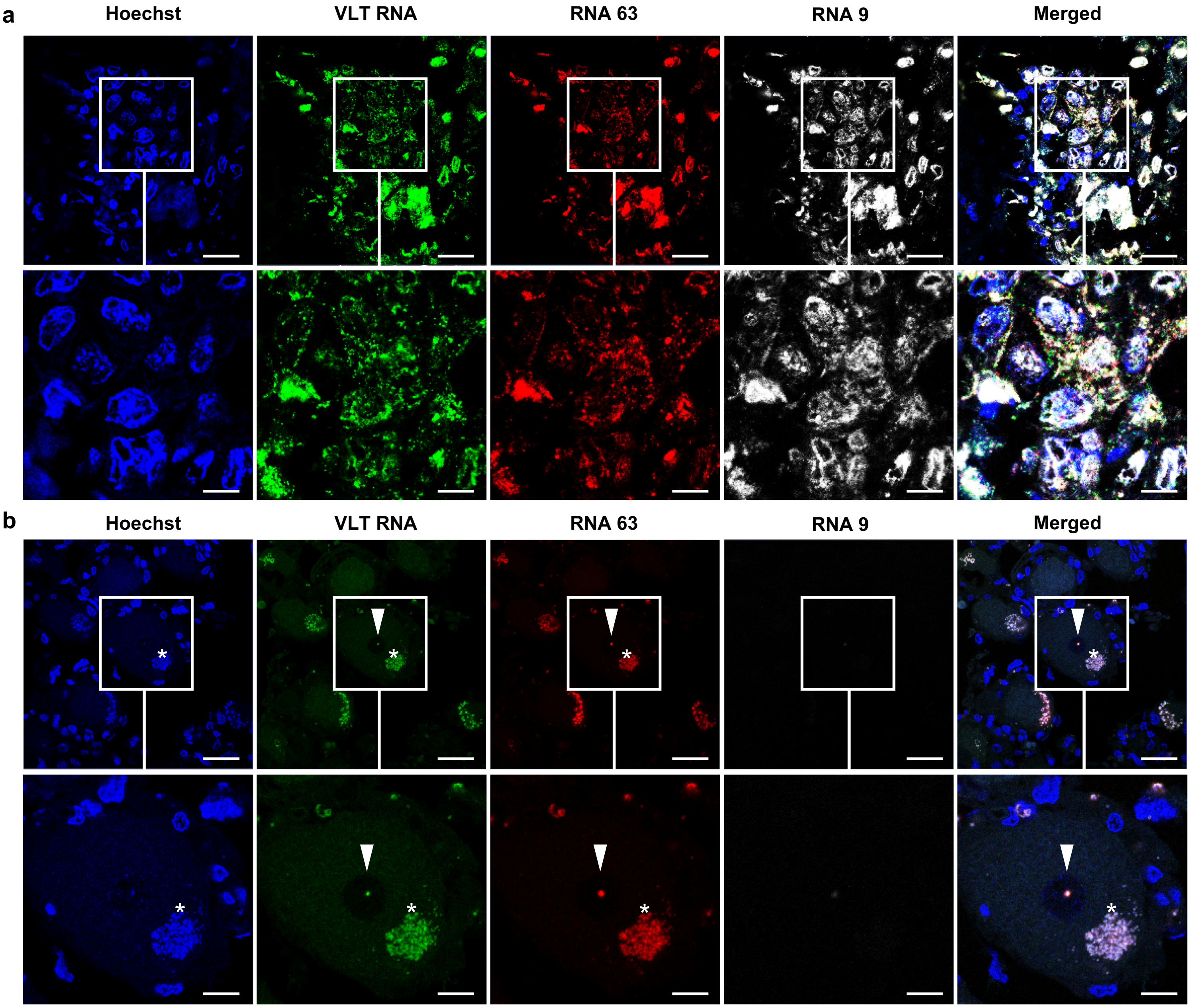

Supplementary Figure 3. Detection of VZV RNA 9, RNA 63 and VLT RNA by fluorescent multiplex in situ hybridization. Detection of VZV VLT, RNA 9 and RNA 63 transcripts by multiplex fluorescent in situ hybridization (ISH) on **a** human zoster skin biopsy and **b** human trigeminal ganglia (TG). Lower row shows enlargement of area indicated by the white box in the upper row. Scale bars: 25 µm (upper rows) and 10 µm (lower rows). **b** Representative images are shown for n=5 human TG analyzed. Asterisks indicate autofluorescent lipofuscin granules in neurons. Arrowheads indicate RNA 63 (red) and VLT (green) ISH signal.

pVLT

MPRLLRDRIA GIPNRVRTYQ GAVFTPWVPD IPTLTTNSNT QILDDHGSPA PRSGVAVQIQ SSHTPPGSPI EQQDGLHWTP AERTLDAGGG PCPNTNKAEV VQTRHGFSEI GNGAHAYGAD KERYEDISPP PCNTRK

pORF63

MFCTSPATRG DSSESKPGAS VDVNGKMEYG SAPGPLNGRD TSRGPGAFCT PGWEIHPARL VEDINRVFLC IAQSSGRVTR DSRRLRRICL DFYLMGRTRQ RPTLACWEEL LQLQPTQTQC LRATLMEVSH RPPRGEDGFI EAPNVPLHRS ALECDVSDDG GEDDSDDDGS TPSDVIEFRD SDAESSDGED FIVEEESEES TDSCEPDGVP GDCYRDGDGC NTPSPKRPQR AIERYAGAET AEYTAAKALT ALGEGGVDWK RRRHEAPRRH DIPPPHGV

pVLT-ORF63

MPRLLRDRIA GIPNRVRTYQ GAVFTPWVPD IPTLTTNSNT QILDDHGSPA PRSGVAVQIQ SSHTPPGSPI EQQDGLHWTP AERTLDAGGG PCPNTNKAEV VQTRHGFSEI GNGAHAYGAG FVRFITRQRR VGFKGKGYYG PKDMFCTSPA TRGDSSESKP GASVDVNGKM EYGSAPGPLN GRDTSRGPGA FCTPGWEIHP ARLVEDINRV FLCIAQSSGR VTRDSRRLRR ICLDFYLMGR TRQRPTLACW EELLQLQPTQ TQCLRATLME VSHRPPRGED GFIEAPNVPL HRSALECDVS DDGGEDDSDD DGSTPSDVIE FRDSDAESSD GEDFIVEEES EESTDSCEPD GVPGDCYRDG DGCNTPSPKR PQRAIERYAG AETAEYTAAK ALTALGEGGV DWKRRRHEAP RRHDIPPPHG V

pORF63-N+

MDCTGHRQRG HWTLVEVHAR IQTKQKSCKH GMVFPRSETV LMHMVQIKSD TKTFLHPPVI PVNKGFVRFI TRQRRVGFKG KGYYGPKDMF CTSPATRGDS SESKPGASVD VNGKMEYGSA PGPLNGRDTS RGPGAFCTPG WEIHPARLVE DINRVFLCIA QSSGRVTRDS RRLRRICLDF YLMGRTRQRP TLACWEELLQ LQPTQTQCLR ATLMEVSHRP PRGEDGFIEA PNVPLHRSAL ECDVSDDGGE DDSDDDGSTP SDVIEFRDSD AESSDGEDFI VEEESEESTD SCEPDGVPGD CYRDGDGCNT PSPKRPQRAI ERYAGAETAE YTAAKALTAL GEGGVDWKRR RHEAPRRHDI PPPHGV

Supplementary Figure 4. In silico translation of VLT, canonical RNA 63 and VLT-ORF63 fusion transcripts. Amino acid (aa) sequence of VLT protein (pVLT; encoded by VLT or VLT63-2), ORF63 protein (pORF63; encoded by RNA 63-1 or VLT63-3), VLT-ORF63 protein (pVLT-ORF63; encoded by VLT63-1) and pORF63-N+ (encoded by VLT63-2). The sequence of 24 aa peptide used as immunogen to generate the chicken anti-pVLT-ORF63 polyclonal antibody is highlighted in grey color.

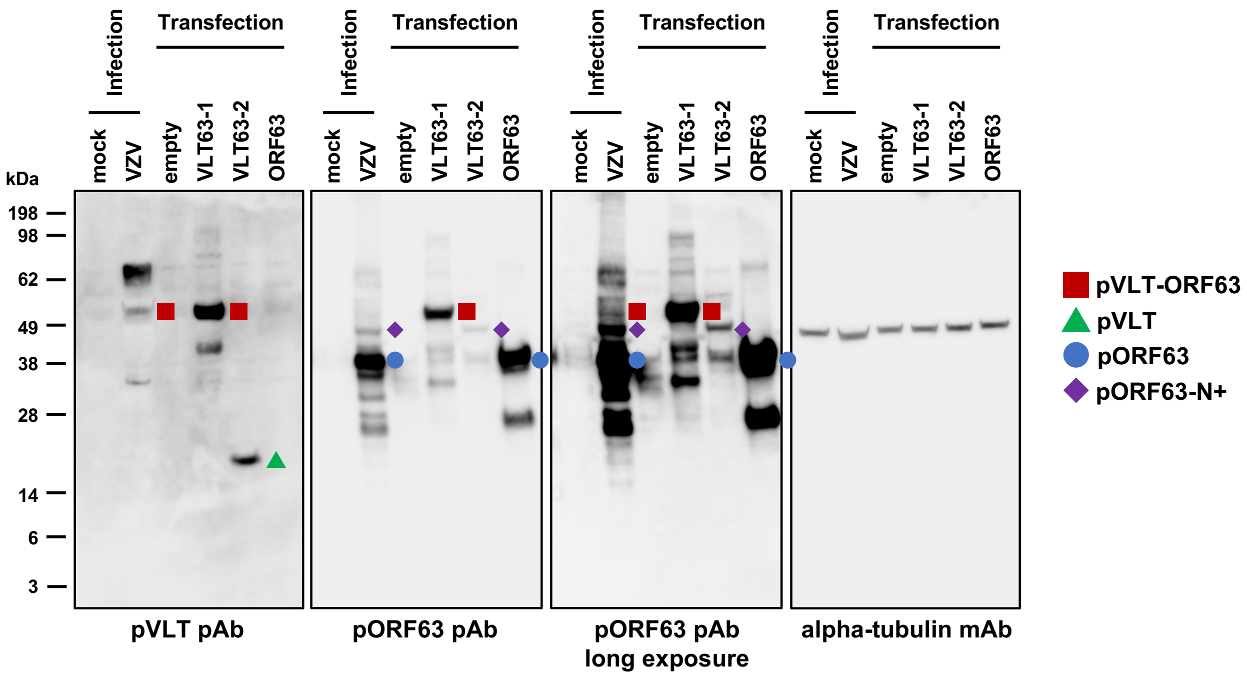

Supplementary Figure 5. Protein coding potential of VLT-ORF63 fusion transcripts.
Immunoblotting analysis using antibodies directed to pVLT and pORF63 in the context of mock- or VZV-infection in CS-CA-empty or CS-CA-VZV transfected ARPE-19 cells. Red squares indicate pVLT-ORF63 (45.989-kDa), green triangles indicate pVLT (14.728-kDa), blue circle indicates pORF63 (30.494-kDa) and purple diamonds indicate pORF63-N+ (40.723-kDa). Images are representative of two independent experiments. Molecular weight marker (kDa) is shown in left.

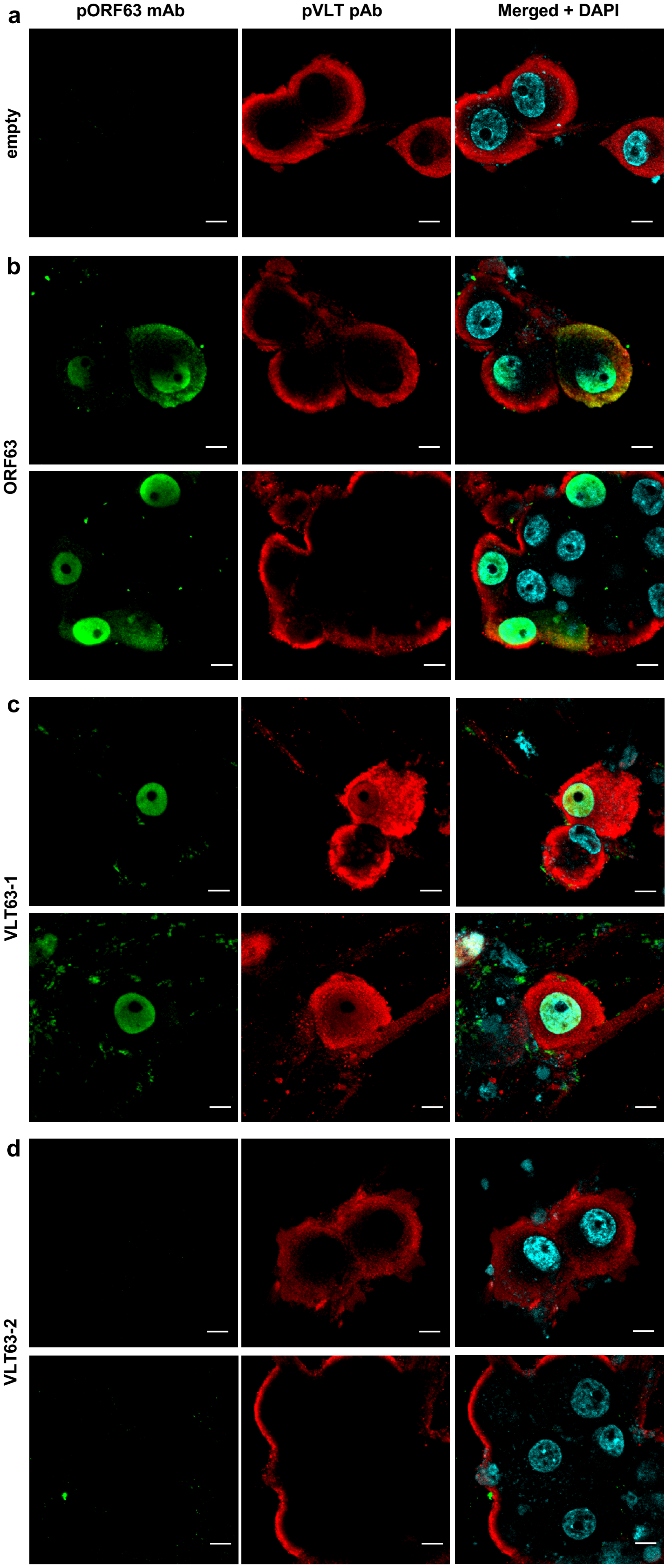

Supplementary Figure 6. Transduced HSN express ectopic proteins encoded by replication incompetent lentivirus vectors. Confocal analysis using antibodies against pORF63 and pVLT in human iPSC-derived sensory neurons (HSN) transduced with replication incompetent lentivirus vectors encoding the following ORFs: **a** no gene (empty), **b** ORF63, **c** VLT63-1 or **d** VLT63-2. HSN cultures were maturated for 49 days and transduced with each vector for 14 days. The signal obtained with anti-pORF63 mAb staining was observed in the nucleus of both ORF63- and VLT63-1-transduced HSN, in cytoplasm of ORF63-transduced HSN, but undetectable in HSN transduced with empty vector or VLT63-2. The anti-pVLT pAb showed a nonspecific cytoplasmic signal in all transduced HSN, including ‘empty’, and was too strong to determine if pVLT alone is expressed by VLT63-2 transduction. Nuclear specific signal by anti-pVLT pAb was only detected in VLT63-1-transduced HSN and co-localized with the pORF63 signal, indicating nuclear expression of pVLT-ORF63. Images are representative of two independent experiments. Scale bar: 10 μm. ORF; open reading frame, mAb; monoclonal antibody, pAb; polyclonal antibody.

Supplementary Table 1. Primers for qPCR assay.

Target	Name	Sequence (5' → 3')
beta-actin	beta-actinF961	GCA CCC AGC ACA ATG AAG A
	beta-actinR1024	CGA TCC ACA CGG AGT ACT TG
VLT, VLT-RNA 63	VLTexonA101426F	CAA CGG AGT GTC GTC TTG GA
	VLTexon1F102413	GGC ATT TTA AAC GGG TCC GG
	VLTexon2F102837	CGA GAC CGG ATT GCG GGC AT
	VLTexon3F103794	TGG ACG ATC ACG GTA GTC CT
	VLTexon4F104342	AAC ACG GCA TGG TTT TTC CG
	VLTexon5F104763	ACG AAG ACA TTT CTC CAC CCC
	VLTexon1R102394	CCG GAC CCG TTT AAA ATG CC
	VLTexon2R102864	CCC TGG TAA GTC CGT ACA CG
	VLTexon3R103847	ATT GAA TCT GCA CAG CAA CCC
	VLTexon4R104361	CGG AAA AAC CAT GCC GTG TT
	VLTexon5R104778	ACC CTC GAG TAC GGG TAT TAC AGG G
	ORF63R19	CCG GTG AGG TGC AAA ACA TG
RNA 4	ORF4F363	GGG GAC ATC GAC GAT CAT CC
	ORF4R415	GTA GGA CGC CGT CTT CGA TT
RNA 9	ORF9F224	AAA AAT ACG ACC CCT CGC GT
	ORF9R298	TCA TGT CTC AAA CGG GCC TC
RNA 16	ORF16F1091	GGA AAC TCC CCG AAA CCA
	ORF16R1158	CAC TGG AGG AGC CAC ACA A
RNA 29	ORF29F2381	GCC TTG CAA GTG CGT ACC
	ORF29R2440	CTA GGG CCC CGT GTA ACA TA
RNA 31	ORF31F331	CAG GAC GCC GAA ACA AAA
	ORF31R393	TAC GAT TGT GGA GCC TGT TG
RNA 49	ORF49F21	CGG TCG AGG AGG AAT CTG TG
	ORF49R80	CCG TTG CAC GTA ACA AGC TC
RNA 61	ORF61F150	CAG CGT CCA GTG TCC TCT CT
	ORF61R210	ACT TAC GAT CTT ATG CAG GAT GG
RNA 62	ORF62F2016	TCC ACC GGA TGA TCG TTT AC
	ORF62R2083	GGA GGC TTC TGC TCT CGA C
RNA 63(cds)	ORF63F556	TCG GAC GGG GAA GAC TTT AT
	ORF63R622	CGT CTG GTT CAC AAG AAT CG
RNA 63(utr)	ORF63up100F	AAC GTT TGG GTG TGT GTT TTG T
	ORF63up1R	GTC CTT GGG GCC GTA GTA AC
RNA 66	ORF66F688	TTC CCC GTG GAT ATT AAT GC
	ORF66R752	GGA GAG TTT GTG GCG ATT GT
RNA 68	ORF68F661	TTA AAA CAT ACA ACA TGC TTT CAA GA
	ORF68R720	AGT ATT TTC CGC GCA ATC C
CS-CA vector	CSCA1831F	CAA CTC ACA GTC TGG GGC AT
	CSCA1969R	TAG CAT TCC AAG GCA CAG CA

Supplementary Table 2. Clinical features of human trigeminal ganglia donors used for RNA extraction.

Donor[a]	Age	Gender[b]	Cause of death	Neurological Disease	PMI[c] (hr:min)
1 : s07/122	94	F	Cerebrovascular accident	Non-demented control	4:05
2 : s09/066	99	F	unknown	Non-demented control	4:15
3 : s09/185	70	M	Cachexia and dehydration	Alzheimer’s disease	4:00
4 : s10/349	95	F	Cachexia	Alzheimer’s disease	4:30

[a]Donor number in this study and corresponding Netherlands Brain Bank number.
[b] F, female; M, male. [c]PMI: post-mortem interval.

Supplementary Table 3. Clinical features of human trigeminal ganglia donors used for ISH.

Donor[a]	Age	Gender[b]	Cause of death	Neurological Disease	PMI[c] (hr:min)
s06/235	80	F	Cardiac arrest	Bipolar disorder	9:30
s11/084	63	F	Gastrointestinal bleeding	Frontotemporal dementia; Pick's disease	4:00
s11/088	81	F	Cachexia and dehydration	Alzheimer's disease	3:35
s11/092	64	F	Cachexia	Frontotemporal dementia; tauopathy	7:30
s11/097	90	M	Cardiac insufficiency	Lewy body dementia	4:05
s13/010	89	F	Heart failure with dehydration	Non-demented control	6:35
s13/013	89	M	Urosepsis	Non-demented control	6:50

[a]Donor number in this study and corresponding Netherlands Brain Bank number.
[b] F, female; M, male. [c]PMI: post-mortem interval.

Supplementary Table 4. Primers for cDNA cloning and 5'-RACE analysis.

Target	Name	Sequence (5' → 3')
**cDNA cloning**		
CS-CA-MCS linearization	CSCAInFusionF	AGA TAT CCA GCA CAG TGG CG
	CSCAInFusionR	CGT TGCCCA GGA GCT GTA GG
CS-CA-ORF63	ORF63up20xhoF	ACC CTC GAG GTT ACT ACG GCC CCA AGG
	ORF63xhoR	ACC CTC GAG CTA CAC GCC ATG G
CS-CA-VLT-ORF63	InFusionCSCAVLTcoreTSSF	CCT ACA GCT CCT GGG CAA CGG CAG ACT ATC CAG TTG GCA
	InFusionCSCAORF63R	CGC CAC TGT GCT GGA TAT CTC TAC ACG CCA TGG
**5'-RACE analysis**		
First-strand cDNA synthesis	SMARTerIIA Oligonucleotide	AAG CAG TGG TAT CAA CGC AGA GTA CAT GGG XXXXX (X :=undisclosed base)
	5'-RACE CDS Primer A	$(T)_{25}$VN (V=A, G or C, N=A, C, G or T)
5'-RACE PCR	Universal Primer Long	CTA ATA CGA CTC ACT ATA GGG CAA GCA GTG GTA TCA ACG CAG AGT
	Universal Primer Short	CTA ATA CGA CTC ACT ATA GGG C
	(InFusion)VLTexon4R104361	GAT TAC GCC AAG CTT CGG AAA AAC CAT GCC GTG TT
	(InFusion)VLTexon5R104799	GAT TAC GCC AAG CTT GTT TGT GGA CTT ACC TTT ATT TAC G
	(InFusion)ORF63R622	GAT TAC GCC AAG CTT CGT CTG GTT CAC AAG AAT CG
	(InFusion)ORF63R805	GAT TAC GCC AAG CTT GGC GCG GGG CTT CGT GTC GA
pRACE linearization	pRACE-F	AAG CTT GGC GTA ATC ATG GTC
	pRACE-R	AGT GAG TCG TAT TAG GAA TTC AC
Sequencing	M13forward	GCC GCT GTA AAA CGA CGG CCA GT
	M13reverse	GGC CGC AGG AAA CAG CTA TGA CC
