# Supplementary material for: Varicella-zoster virus VLT-ORF63 fusion transcript induces broad viral gene expression during reactivation from neuronal latency
Source: Nat Commun. 2020 Dec 10;11:6324. doi: 10.1038/s41467-020-20031-4 (PMC7730162; doi:10.1038/s41467-020-20031-4)
Supplement: Supplementary file 3 — Reporting Summary [file 41467_2020_20031_MOESM3_ESM.pdf]

nature research

Corresponding author(s): Tomohiko Sadaoka

Last updated by author(s): Oct 27, 2020

Reporting Summary

Nature Research wishes to improve the reproducibility of the work that we publish. This form provides structure for consistency and transparency in reporting. For further information on Nature Research policies, see Authors & Referees and the Editorial Policy Checklist .

Statistics

For all statistical analyses, confirm that the following items are present in the figure legend, table legend, main text, or Methods section.

n/a	Confirmed	
☐	☒	The exact sample size (*n*) for each experimental group/condition, given as a discrete number and unit of measurement
☐	☒	A statement on whether measurements were taken from distinct samples or whether the same sample was measured repeatedly
☒	☐	The statistical test(s) used AND whether they are one- or two-sided<br>*Only common tests should be described solely by name; describe more complex techniques in the Methods section.*
☒	☐	A description of all covariates tested
☒	☐	A description of any assumptions or corrections, such as tests of normality and adjustment for multiple comparisons
☒	☐	A full description of the statistical parameters including central tendency (e.g. means) or other basic estimates (e.g. regression coefficient) AND variation (e.g. standard deviation) or associated estimates of uncertainty (e.g. confidence intervals)
☒	☐	For null hypothesis testing, the test statistic (e.g. *F*, *t*, *r*) with confidence intervals, effect sizes, degrees of freedom and *P* value noted<br>*Give P values as exact values whenever suitable.*
☒	☐	For Bayesian analysis, information on the choice of priors and Markov chain Monte Carlo settings
☒	☐	For hierarchical and complex designs, identification of the appropriate level for tests and full reporting of outcomes
☒	☐	Estimates of effect sizes (e.g. Cohen's *d*, Pearson's *r*), indicating how they were calculated

Our web collection on statistics for biologists contains articles on many of the points above.

Software and code

Policy information about availability of computer code

Data collection	Sequence read data generated on the nanopore minION were captured using MinKnow v3.1.9, the freely available Oxford Nanopore Technologies command software.
Data analysis	Sequence read data were subsequently processed using freely available software including Guppy v3.2.2, SAMtools v1.9, BEDtools v2.27.1, minimap2 v2.15, and the R packages BioConductor v3.11 and Gviz v1.3.2

For manuscripts utilizing custom algorithms or software that are central to the research but not yet described in published literature, software must be made available to editors/reviewers. We strongly encourage code deposition in a community repository (e.g. GitHub). See the Nature Research guidelines for submitting code & software for further information.

Data

Policy information about availability of data

All manuscripts must include a data availability statement. This statement should provide the following information, where applicable:

- Accession codes, unique identifiers, or web links for publicly available datasets
- A list of figures that have associated raw data
- A description of any restrictions on data availability

Note that the following text is also included after the Methods section of the main text.
Basecalled fast5 nanopore dRNA- and cDNA-Seq datasets generated as part of this study can be downloaded from the European Nucleotide Archive (ENA) under the following study accession: PRJEB36978 (https://www.ncbi.nlm.nih.gov/sra/?term=PRJEB36978). Source data are provided with this paper or are available from the authors upon request.

Field-specific reporting

Please select the one below that is the best fit for your research. If you are not sure, read the appropriate sections before making your selection.

- [x] Life sciences
- [ ] Behavioural & social sciences
- [ ] Ecological, evolutionary & environmental sciences

For a reference copy of the document with all sections, see nature.com/documents/nr-reporting-summary-flat.pdf

Life sciences study design

All studies must disclose on these points even when the disclosure is negative.

Sample size	No sample size calculation was performed. Sample size was followed the standard in the field.<br>Sample size for human specimens was dictated by the availability of materials and experimental costs.<br>A sample size two to six was used for biological replicates (specified in the figure legends) for all in vitro experiments except nanopore cDNA-seq due to the limited amount of purified RNA. For nanopre cDNA-seq, RNAs from two biological replicates were combined and sequenced. The same RNAs were independently analyzed by RT-qPCR and confirmed their consistency between two biological replicates (data are listed).
Data exclusions	No data were excluded from the analysis.
Replication	All experiments made use two to six biological replicates that were reliably reproducible.
Randomization	No group allocation was involved in this study, and all samples were treated identically, so this was not needed.
Blinding	Investigators were not blinded during experiments. Sample preparation, collection and analyses were performed by the same researcher. All samples were treated and analyzed in a similar procedure.

Reporting for specific materials, systems and methods

We require information from authors about some types of materials, experimental systems and methods used in many studies. Here, indicate whether each material, system or method listed is relevant to your study. If you are not sure if a list item applies to your research, read the appropriate section before selecting a response.

Materials & experimental systems

n/a	Involved in the study	
☐	☒	Antibodies
☐	☒	Eukaryotic cell lines
☒	☐	Palaeontology
☒	☐	Animals and other organisms
☐	☒	Human research participants
☒	☐	Clinical data

Methods

n/a	Involved in the study	
☒	☐	ChIP-seq
☒	☐	Flow cytometry
☒	☐	MRI-based neuroimaging

Antibodies

Antibodies used	Primary antibodies<br>Chicken anti-VZV pVLT-ORF63 polyclonal antibody (custom-made by Cosmo Bio) , Rabbit anti-VZV pVLT polyclonal antibody (custom-made by Sigma-Aldrich), Rabbit anti-VZV ORF63 polyclonal antibody (custom-made by Sigma-Aldrich), mouse anti-VZV ORF63 monoclonal antibody (clone VZ63.08), mouse anti-VZV gE monoclonal antibody (clone 9), anti-mouse alpha-tubulin monoclonal antibody (clone B-5-1-2, Sigma-Aldrich), sheep anti-NGF polyclonal antibody (cat# AB1528SP, EMD Millipore).<br>Secondary antibodies<br>Alexa Fluor 488- or Alexa Fluor 647-conjugated donkey anti-mouse IgG (cat# A21202 or A31571, Thermo Fisher Scientific), Alexa Fluor 594-conjugated donkey anti-rabbit IgG (cat# A21207, Thermo Fisher Scientific), Alexa Fluor 488-conjugated donkey anti-chicken IgY (Jackson ImmunoResearch Laboratories), anti-mouse IgG HRP-linked Whole Ab Sheep and anti-rabbit IgG HRP-linked Whole Ab Donkey (cat# NA931 and NA934, GE Healthcare Bio-Sciences).
Validation	All VZV antibodies were validated using each gene expressing plasmid-transfected cells and in VZV-infected cells by IB or IF. The specifications of commercially available antibodies can be found on the manufacture's website using their catalogue numbers.

Eukaryotic cell lines

Policy information about cell lines

Cell line source(s)	ARPE-19 cells were obtained from ATCC (CRL-2302).<br>HEK-293T cells were obtained from ATCC (CRL-3216).<br>Human iPSC-derived sensory neuron progenitors were purchased from Axol biosciences.
Authentication	ARPE-19 and HEK-293T cells were obtained from the ATCC, no further cell line authentication method was used.<br>Human iPSC-derived sensory neurons were confirmed morphologically and by staining with multiple sensory neuronal markers after terminal differentiation from human iPSC-derived sensory neuron progenitors.
Mycoplasma contamination	ARPE-19 and HEK-293T cells were confirmed to be negative for mycoplasma contamination.<br>Human iPSC-derived sensory neuron progenitors were tested and confirmed to be negative for mycoplasma contamination by the supplier (Axol biosciences).
Commonly misidentified lines<br>(See ICLAC register)	No commonly misidentified lines were used.

Human research participants

Policy information about studies involving human research participants

Population characteristics	Population characteristics are described in Supplementary Tables 2 and 3.
Recruitment	Postmortem human trigeminal ganglion specimens were obtained from the Netherlands Brain Bank. All donors had provided written informed consent for brain autopsy and the use of material and clinical information for research purposes. All donors analyzed contained VZV DNA in their ganglia and were included in the study; no further selection criteria were used.
Ethics oversight	VU University Medical Center, Amsterdam, project number 2009/148, and Kobe University, Kobe, project number 170107

Note that full information on the approval of the study protocol must also be provided in the manuscript.
